# Information Load from Neuromediator Diffusion to Extrasynaptic Space: The Interplay between the Injection Frequency and Clearance

**DOI:** 10.3390/biology13080566

**Published:** 2024-07-26

**Authors:** Andrey Shuvaev, Olga Belozor, Anton Shuvaev

**Affiliations:** 1 Institute of Fundamental Biology and Biotechnology, Siberian Federal University, 660041 Krasnoyarsk, Russia; shuvaevan@krasgmu.ru; 2 Research Institute of Molecular Medicine and Pathobiochemistry, Krasnoyarsk State Medical University, 660022 Krasnoyarsk, Russia; olsbelor@gmail.com

**Keywords:** glutamate, diffusion, mutual information

## Abstract

**Simple Summary:**

We investigate how glutamate spillover affects information transfer by analyzing the mutual information between release and extrasynaptic locations. Through tetanic release simulations, we aim to quantify the efficiency of information transfer in the presence of excess glutamate. Our study seeks to offer insights into synaptic information processing and excitotoxicity.

**Abstract:**

In our study, we simulate the release of glutamate, a neurotransmitter, from the presynaptic cell by modeling the diffusion of glutamate into both synaptic and extrasynaptic space around the synapse. We have also incorporated a new factor into our model: convection. This factor represents the process by which the body clears glutamate from the synapse. Due to this process, the physiological mechanisms that typically prevent glutamate from spreading beyond the synapse are altered. This results in a different distribution of glutamate concentrations, with higher levels outside the synapse than inside it. The variety of biological effects that occur in response to this extrasynaptic glutamate highlights the importance of preventing neurotransmitters from spreading beyond the synapse. We aim to explain the physical reasons behind these biological effects, which are observed as excitotoxicity. Our results show that preventing the spread of glutamate outside the synapse increases the amount of information exchanged within the synapse and its surroundings for frequencies of glutamate release up to 30–50 Hz, followed by a decrease. Additionally, we find that the rate at which glutamate is cleared from the synapse is effective at relatively low levels (≤0.5 nm/μs in our calculation grid) and remains constant at higher levels.

## 1. Introduction

Glutamate plays a key role in signaling in the brain. However, unlike synaptic glutamate, the action of extrasynaptic glutamate can damage or even kill postsynaptic neurons, a phenomenon known as excitotoxicity [1]. This effect is mainly caused by extrasynaptic N-methyl-D-aspartate receptors (NMDARs), which respond to exogenous glutamate [2]. The activation of NMDARs alters neuronal excitability and signaling strength. Recent studies [3] have shown that the structure of the extracellular space can alter under pathological conditions, leading to disturbances in neuronal activity. In such cases, NMDAR currents are influenced by the intracleft diffusion coefficient of glutamate.

Another source of excitotoxicity is the postsynaptic metabotropic glutamate receptor (mGluR), which also binds with glutamate [4]. These receptors are primarily located on the edges of synapses and in the extrasynaptic zone [5]. In cerebellar pathology, such as spinocerebellar ataxia (SCA), slow cation influx through mGluR1-dependent TRPC channels is the main source of excitotoxicity [6].

Both NMDAR and mGluR pathways activated by glutamate alter the calcium flux inside neurons. The excessive amount of calcium triggers apoptotic changes in the cell [7]. This excess is expected due to the increased total concentration of glutamate, whether acute or chronic. However, the increased concentration inside the synapse does not lead to harmful calcium activity. Apoptosis occurs when extrasynaptic entry is detected due to increased glutamate in pathological conditions [8,9]. In the synapse, there are two main mechanisms that prevent glutamate from spilling over: the higher density of the medium and faster clearance of glutamate outside the synapse [10]. The primary role in glutamate reuptake is carried out by astrocytes, which have three effects: buffering glutamate excess (rapidly taking up excess glutamate to prevent its accumulation in the synaptic cleft and extracellular space), spatial buffering, and synaptic isolation (positioning around synapses and efficiently clearing glutamate from critical areas to maintain homeostasis), as well as regulating synaptic strength and plasticity.

Nevertheless, even a small amount of neurotransmitter outside the synaptic cleft can cause hyperreaction of the postsynaptic cell [11]. Moreover, glutamate spillover triggers the repetitive secondary release of glutamate from the presynaptic cell [12].

These facts highlight the physiological mechanisms for preventing excitotoxicity. It is still unclear whether excitotoxicity is a consequence of high levels of glutamate or whether neurons hyperreact to normal concentrations. Treatment efforts have shown that excitotoxicity is not always relieved by suppressing neuron hyperactivity or by the drug effects on glutamatergic neurotransmission [13,14].

Among other factors, the clearance of glutamate has been demonstrated to directly impact synaptic plasticity [15]. This link explores the impact of information transfer and processing on the response of the postsynaptic cell. Moreover, information transmission is linked to peak frequency, synaptic type, and synaptic plasticity [16]. Synaptic plasticity, such as long-term potentiation (LTP) and long-term depression (LTD), is a key process for learning and memory and is highly sensitive to the concentration and clearance rate of glutamate. Disruption in these processes due to inadequate glutamate clearance can lead to pathological conditions, including neurodegenerative diseases.

In our study, we are investigating the information load by examining the mutual information between the release location and extrasynaptic location while varying the conditions of spillover. The excessive amount of glutamate is simulated by tetanic release at various frequencies. By analyzing the mutual information, we aim to quantify the efficiency of information transfer in the presence of glutamate spillover. Understanding how spillover impacts signaling fidelity can provide insights into the mechanisms of excitotoxicity and help develop therapeutic strategies to mitigate the detrimental effects of excessive glutamate. Ultimately, the aim of this study is to offer a thorough understanding of the connection between synaptic glutamate dynamics, information processing, and neuronal health.

## 2. Model

### 2.1. Glutamate Diffusion

The majority of existing models of neurotransmitter diffusion are confined to the synapse and/or operate with a constant diffusion coefficient [17,18,19]. For predicting the concentration of glutamate in the extrasynaptic space, which has different geometry and constants from the synaptic space, we needed a suitable model. The ideal candidate for this purpose is the Barbour model [20], as it incorporates both synaptic and extrasynaptic spaces with complex geometrical transitions. It uses a composite volume function to represent the complex transitions from the synaptic cleft to the extracellular space, providing a more intricate spatial representation.

The Barbour’s model is supplemented with the clearance term Ω, which represents the process of glutamate binding to receptors, its removal by astrocytes, and degradation. The final equation we use can be written as:

(1) $$\begin{array}{ccc} \frac{\partial c(r,t)}{\partial t} & = & \nabla \cdot \left(D(r)\nabla c(r,t)-\Omega (r)c(r,t)\right), \end{array}$$

where c(r,t) represents the glutamate concentration or number of molecules at location *r* at time *t*, ∇c(r,t) denotes the spatial gradient of *c*, and (∇·) represents the divergence operator.

**Figure 1:**
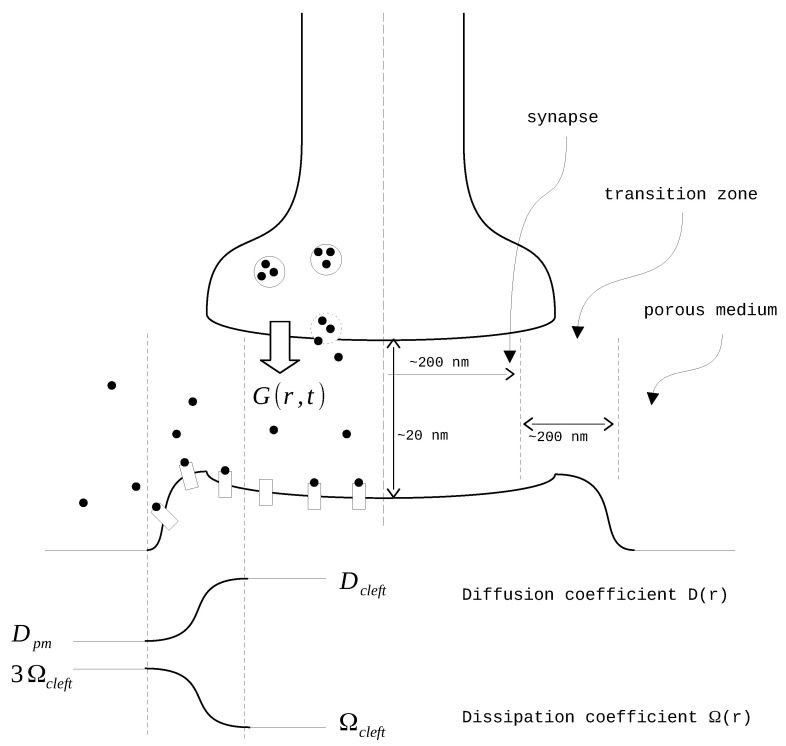
The synapse scheme.

The diffusion coefficient D(r) and the rate of clearance Ω(r) vary depending on the distance from the synapse center as suggested in [20] (Figure 1). The modeling domain is divided into three parts: the synapse itself, the transition zone, and the extrasynaptic space. In the synapse zone, both *D* and Ω have constant values. In the transition zone, these values are interpolated using a sixth-order polynomial function f(r). The coefficients of this polynomial are derived from the conditions of equality to zero on the side of the synapse and to one on the outer side. Additionally, the first and second derivatives of the polynomial are set to zero on both sides. After the interpolation, the final smooth functions take the form of $X\left(r\right)=X_{cleft}+f\left(r\right)\left(X_{pm}-X_{cleft}\right)$ where *X* represents either *D* or Ω:

$$D\left(r\right)=\left\{\begin{array}{c} D_{cleft}\;\left(r\leq R_{cleft}\right)\\ D_{cleft}+f\left(r\right)\left(D_{pm}-D_{cleft}\right)\;\left(R_{cleft}<r<R_{pm}\right)\\ D_{pm}\;\left(r\geq R_{pm}\right) \end{array}\right.\;,$$

$$\Omega \left(r\right)=\left\{\begin{array}{c} \Omega_{cleft}\;\left(r\leq R_{cleft}\right)\\ \Omega_{cleft}+f\left(r\right)\left(\Omega_{pm}-\Omega_{cleft}\right)\;\left(R_{cleft}<r<R_{pm}\right)\\ \Omega_{pm}\;\left(r\geq R_{pm}\right) \end{array}\right.\;.$$

The model equations are solved using cylindrical coordinates. We assume isotropic conditions based on the angle of neurotransmitter spreading and the negligible height of the synapse. In that case, the general Equation (1) transforms into:

(2) $$\begin{array}{ccc} \frac{\partial c(r,t)}{\partial t}+\frac{\partial G(r,t)}{\partial t} & = & D\left(r\right)\frac{\partial^{2}c\left(r,t\right)}{\partial r^{2}}+\left(\frac{\partial D(r)}{\partial r}+\frac{D(r)}{r}-\Omega \left(r\right)\right)\frac{\partial c(r,t)}{\partial r}-\\ & & \left(\frac{\partial \Omega (r)}{\partial r}+\frac{\Omega (r)}{r}\right)c\left(r,t\right), \end{array}$$

where G(r,t) represents the tetanic release of glutamate to the synapse. It is modeled as a Gaussian source $G\left(r,t\right)=N\left(r;\mu ,\sigma \right)\cdot \delta \left(t-t^{\prime }\right)$, with $t^{\prime }$ representing the time of the impulse. The parameters of the normal distribution are set as follows: μ=0 nm and $\sigma =0.68\cdot R_{cleft}$ nm (see Table 1). Since the time of the first impulse is set to t=0, the initial condition for the model would be G(r,0)=N(r;μ,σ). The boundary condition for the model is set as c(0,t)=0.

Numerical routine. To solve Equation (2), we employ the finite differences method. When calculating the derivative of the delta function with respect to time in the expression for $\frac{\partial G(r,t)}{\partial t}$, we approximate it using a narrow normal distribution $\delta \left(t-t^{\prime }\right)\approx N\left(t,t^{\prime },\sigma_{t}\right)$, where $\sigma_{t}\to 0$. This allows us to obtain $\frac{\partial G(r,t)}{\partial t}=\frac{2}{\sigma_{t}^{2}}\left(t^{\prime }-t\right)\cdot N\left(t,t^{\prime },\sigma_{t}\right)$.

To calculate the boundary condition at r=0, we apply l’Hôpital’s rule to the uncertainties: $\frac{D_{cleft}}{r}\frac{\partial c(r,t)}{\partial r}=D_{cleft}\frac{\partial^{2}c\left(r,t\right)}{\partial r^{2}}$ and $\frac{\Omega_{cleft}}{r}c\left(r,t\right)=\Omega_{cleft}\frac{\partial c(r,t)}{\partial r}$. This results in

(3) $${\left.\frac{\partial c(r,t)}{\partial t}\right|}_{r=0}=2D_{cleft}{\left.\frac{\partial^{2}c\left(r,t\right)}{\partial r^{2}}\right|}_{r=0}-2{\left.\Omega_{cleft}\frac{\partial c(r,t)}{\partial r}\right|}_{r=0}$$

Impulses are generated within a 200-millisecond time period, and the entire simulation lasted 500 milliseconds.

### 2.2. Propagation of Information in a Synapse

The informational load is calculated as the mutual information (MI) between locations *A* and *B* for the transfer time *T*, according to [21]:

(4) $$\begin{array}{ccc} I(A_{t_{n}},B_{t_{n}+T}) & = & \sum_{n}P\left(A_{t_{n}},B_{t_{n}+T}\right)\log_{2}\frac{P(A_{t_{n}},B_{t_{n}+T})}{P\left(A_{t_{n}}\right)P\left(B_{t_{n}+T}\right)}. \end{array}$$

Here, $P(A_{t},B_{t+T})$ represents the joint probability of finding a specific molecule of neurotransmitter at location *A* during the *n*-th time step, $t_{n}$, and at location *B* at a later time step, $t_{n}+T$. $P(A_{t_{n}})$ and $P(B_{t_{n}+T})$ represent marginal probabilities. Most evaluations are conducted with *A* being located within the postsynaptic density (PSD) and *B* situated in the transition zone.

This equation quantifies how much knowing the state of neurotransmitter molecules at one location A and time $t_{n}$ informs us about their state at another location B and time $t_{n}+T$, considering the probabilistic relationships between these states.

Parameters values are listed in Table 1. The clearance $\Omega_{cleft}$ is free to vary. We utilize a 3-fold difference in the clearance rate between the synaptic cleft and extrasynaptic region: $\Omega_{pm}=3\Omega_{cleft}$ (adapted from [22] ).

## 3. Results

### 3.1. Clearance Transforms the Neurotransmitter Concentration Profile

**Figure 2:**
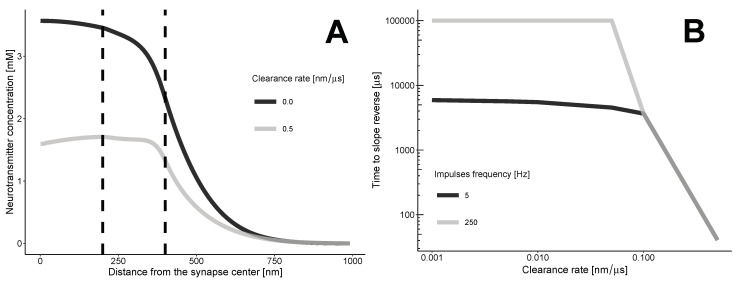
The dynamics of neurotransmitter concentration. (**A**) Without clearance, the concentration of neurotransmitters decreases, resulting in a negative slope. With clearance, the maximum concentration shifts due to convection. At a certain point, the slope reverses. (**B**) The minimal time required for the slope reversal of the neurotransmitter concentration profile. The calculations are based on the approximate Equation (5). The threshold for the clearance rate is 0.1 nm/microsecond. At this value, the minimum time for slope reversal becomes independent of the frequency of impulses.

The transition zone between the synaptic and extrasynaptic spaces creates a barrier that prevents neurotransmitters from spilling over into the synaptic cleft. This barrier, along with the clearance flux, alters the diffusion profile of the neurotransmitter (refer to Figure 2). As the neurotransmitters move along the synapse through convection, at a certain point, the intrasynaptic area starts to contain fewer neurotransmitters per unit distance compared to the transition zone.

The zero-clearance case indicates a gradual decrease in the concentration of neurotransmitters as one moves away from the synapse. The slope of the concentration curve remains negative at any distance and time. The clearance results in a complex, non-monotonic distribution, with both negative and positive slopes, depending on the time and coordinate *r*.

The repeated impulses release neurotransmitters into the synaptic cleft, which can help sustain a gradual decrease. In this case, the time between impulses must be shorter than the time it takes for the profile to become positively sloped.

The time when the profile between the synaptic and extrasynaptic spaces is not monotonic can be calculated using the condition $c\left(r,t^{*}\right){{|}_{r=s_{-}}\leq c\left(r,t^{*}\right)|}_{r=s_{+}}$, where *s* represents the border between the synapse and the transition zone. This condition can be approximated by:

(5) $$c\left(r,t^{*}\right){{|}_{r=A}\leq c\left(r,t^{*}\right)|}_{r=B},$$

where *A* and *B* represent arbitrary points in the synaptic area and transition zone, respectively. At the moment of reversal, there will be an equation instead of an inequality.

Our calculations (Figure 2B) indicate that the clearance of neurotransmitters from the synaptic cleft reduces the profile’s dependence on the frequency of neurotransmitter release. Even a moderate rate of clearance, which we estimated to be 0.1 nanometers per microsecond or higher, leads to a situation where the minimum time for the slope to reverse is not affected by the frequency of impulses.

**Figure 3:**
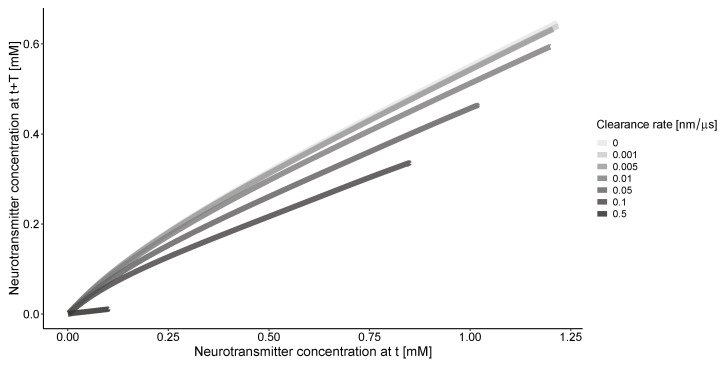
The concentrations of neurotransmitters in the synapse and extrasynaptic space at different time points (*t* = 20 μs, T = 10 μs). Calculations are conducted for an impulse frequency of 50 Hz and various clearance rates. The beginning of the curves on the right side corresponds to the center of synapse at r=0 nm. All curves converge to 0 at the end of the calculation grid when r=1000 nm.

The phase portrait of concentration along the *r* coordinate (Figure 3) indicates that without clearance, there is a consistent relationship between the solutions for the concentration of neurotransmitter at the same location but with a time delay. The clearance process simplifies this relationship. The nonlinearity of the curves is more pronounced at lower clearance rates.

### 3.2. Neurotransmitter Clearance Enhances Mutual Information

**Figure 4:**
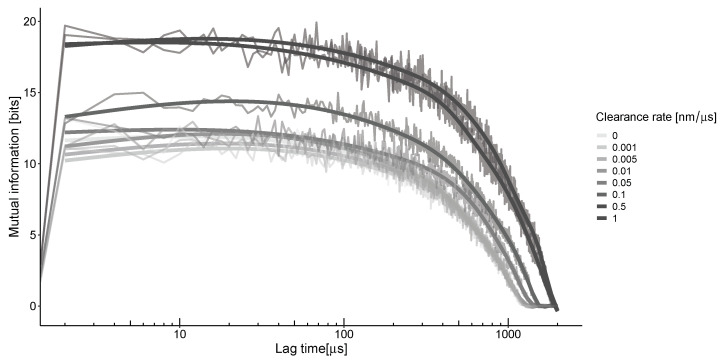
The mutual information between the center of the synapse and the transition zone, which is 300 nm away, at a frequency of 50 Hz. The highest value is observed when there is a ≈40–50-microsecond delay between their locations, regardless of the clearance rates. The thick lines represent the smoothing of the jagged lines of the corresponding color.

To understand the impact of unstable neurotransmitter spread on information transfer, we must consider the concentrations at different locations and times [23]. As the neurotransmitter travels through the synaptic cleft via convection and diffusion, it triggers the opening of channels, a process directly influenced by the concentration of the neurotransmitter. The information that can be obtained from this spread depends on the relative concentrations of molecules in two different parts of the synapse at various points in time. The time difference between these points, known as the lag time, is proportional to the speed at which the neurotransmitter propagates in heterogeneous conditions (Figure 4). The calculation of mutual information along the synaptic distance under these conditions results in characteristic non-monotonic curves. The peak of this curve occurs at the optimal lag time, representing the average time it takes for neurotransmitters to travel from their emission source to the detection point. For the selected parameters and calculation grid, the estimated time is around 50 μs for all clearance rates.

At the same time, the clearance from the cleft reduces the concentration of molecules at a specific location. This, in turn, leads to an increase in information because the occurrence of neurotransmitter release in distant locations during a lag becomes less frequent. We can express this as:

(6) I(X,Y)=H(X)+H(Y)−H(X,Y)=H(Y)−H(Y|X),

where H(X) and H(Y) represent the entropies of events related to finding neurotransmitters in regions *X* and *Y*, respectively. H(X,Y) denotes the joint entropy of these events, while H(Y|X) represents the conditional entropy.

Higher values of I(X,Y) can be achieved by increasing the first two terms in the equation or decreasing the last one. Since the highest entropy corresponds to the uniform distribution, any restriction on the spread of neurotransmitters leads to a narrower distribution and, consequently, a reduction in entropy. For clearances $H\left(X|\Omega =\Omega_{1}\right)>H\left(X|\Omega =\Omega_{2}\right)$ for $\left\{\Omega_{1}<\Omega_{2}\right\}$. Since H(X)+H(Y)≥H(X,Y) due to the non-negativity of mutual information, the decrease in joint entropy is greater than the reduction in individual entropies.

**Figure 5:**
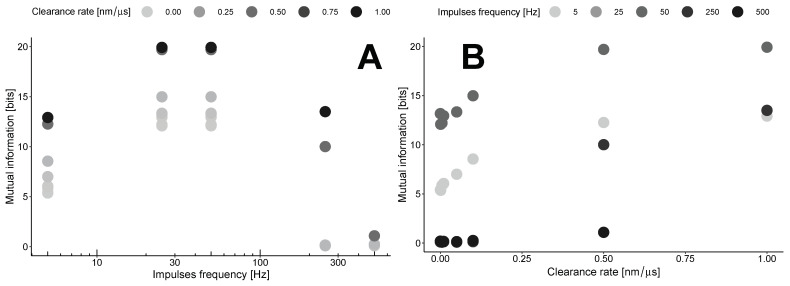
Mutual information as a function of impulse frequency (**A**) or clearance rate (**B**).

This results in the maximization of mutual information by clearance in the area outside the synapses at a specific point in time, known as the lag time. The dependence on the clearance rate (Figure 5B) concerning the impulse frequency shows a gradual increase for low frequencies up to 0.5 nm/μs for most frequencies, followed by a plateau with a different maximum level. The levels of these plateaus are estimated to be around 15–20 bits with the parameters used in our model (Table 1).

These maxima are estimated in the region of the transition from the synaptic to the extrasynaptic space. This point is defined as 300 nm from the center of the synapse disk. Our calculations indicate that in this case, mutual information exhibits nonlinear dynamics with the frequency of impulses (Figure 5A). The optimal frequency for any clearance rate is found to be in the range of 30–50 Hz. At the same time, higher clearance rates indicate a steeper drop from the maximum.

This results in the optimal clearance rate, which represents the trade-off between the efficiency of information transfer and negative biological effects such as excitotoxicity.

High clearance rates (>1.0 nm/μs) are also estimated during stimulation, but they result in unstable outcomes for mutual information. This is due to challenges in accurately calculating the distribution of molecules when their number is relatively small.

Both plots in Figure 5 indicate that the MI increases with higher clearance rates for all frequencies examined in our study. This consistent increase suggests that efficient clearance mechanisms influence the levels of information transfer across the synaptic and extrasynaptic regions, highlighting the delicate balance between neurotransmitter clearance and synaptic signaling efficiency.

Furthermore, our results underscore the importance of considering the biophysical properties of the synaptic environment, such as diffusion coefficients and receptor densities, which can significantly influence the dynamics of neurotransmitter spread and, consequently, the efficiency of information transfer. These findings offer valuable insights into the underlying mechanisms of synaptic communication and may guide the development of therapeutic strategies to reduce excitotoxicity while enhancing synaptic function.

## 4. Discussion

Even a small amount of neurotransmitter in the space outside the synapse can elicit a significant response from the cell. This refers to the clustering and conformational changes in receptors, which result in the spontaneous firing of neurons [24]. Here, we propose a potential physical explanation for these biological changes. The scarcity of glutamate molecules outside the synapse enhances the quantity of information the neuron receives from them. This increase is a result of the clearance of neurotransmitters, which aims to prevent negative effects caused by the spillover of neurotransmitters.

It has been shown that the response of NMDAR depends on the precise concentration of glutamate [25]. The concentration of glutamate is determined by the process of clearance, so the density of receptors on the postsynaptic membrane must also be considered in the regulation process. The density of receptors is highest in the PSD (postsynaptic density) and decreases proportionally as the concentration of neurotransmitter decreases. Normally, this difference is up to 50 times [26], but this ratio can vary due to the ability of receptors to move laterally [27]. The concentration of neurotransmitters may be the primary driving force in this dynamic process. This fact supports the idea that the concentration profile of neurotransmitters determines the distribution of information along the synaptic cleft by influencing the distribution of receptors. The postsynaptic cell can react to high neurotransmitter concentrations either by providing feedback to the presynaptic cell to reduce release or by clustering receptors into nanodomains in regions of high neurotransmitter density as a response to the neurotransmitter profile in the synapse. Since nanodomains are dynamic structures that can change in size, shape, and composition in response to synaptic activity, they can adapt to alterations in the neurotransmitter profile. This adaptation will modify the cell’s sensitivity to synaptic input and modulate synaptic strength and plasticity, ensuring efficient signaling under varying conditions. Experimental data support these theoretical models. For instance, studies have shown that altering the expression or function of glutamate transporters, which are responsible for neurotransmitter clearance, can significantly affect synaptic transmission and plasticity [28].

The mutual information increases with any rate of neurotransmitter clearance, as long as the rate is not zero. At the same time, the clearance does not change the lag time, despite varying clearance rates in different locations. This can be interpreted as a persistent correlation between these points and the effectiveness of clearance in reducing the information load.

At the same time, convection arising from clearance can enhance the transport of neurotransmitters away from the release site more rapidly than diffusion alone, leading to quicker clearance and a broader spatial spread of the neurotransmitter. This can impact how information is encoded and transmitted. Unlike pure diffusion, which tends to create smooth, predictable gradients, convection can produce non-monotonic concentration gradients and more complex patterns of neurotransmitter distribution. This variability can influence how neural signals are interpreted, thereby altering the mutual information between neural signals and neurotransmitter concentration. Moreover, the effects of convection differ between synaptic and extrasynaptic spaces. In synaptic spaces, where neurotransmitter dynamics are tightly regulated, convection might have a smaller impact. In extrasynaptic spaces, convection could play a more significant role in spreading neurotransmitters over larger areas, affecting how signals are integrated and processed.

The unstable behavior of mutual information at high rates is a result of the small number of molecules in two locations. Ergodicity implies that over extended periods, the statistical properties of the neurotransmitter distribution will be uniform across various locations. Hence, the frequency of glutamate release becomes less significant in influencing mutual information once the system reaches equilibrium (30–50 Hz according to our estimation). However, the clear plateau at high rates of glutamate clearance (>0.5 nm/μs) indicates that the clearance does not compensate for the information load from tetanic neurotransmitter release. This could be because the system has reached an optimal state where the neurotransmitter concentration is sufficiently low to prevent excessive spillover, and any additional clearance has a negligible effect on reducing information redundancy.

It is worth mentioning that our model is constrained by the biologically reliable frequency of tetanic stimulation, ranging from tens to hundreds of Hz. Extending the model beyond these limits would necessitate changes to the clearance rate, potentially leading to an overestimation of the impact of high impulse rates on mutual information. Moreover, our model does not consider electric fields, which can affect neurotransmitter movement and significantly influence the predictions. Additionally, no delay between the release of the neuromediator and the initiation of clearance is incorporated into the model.

## 5. Conclusions

In our study, we demonstrated that the efficient clearance of neurotransmitters enhances the amount of information neurons receive by preventing excess spillover, which can potentially cause excitotoxicity. The clearance of neurotransmitters, facilitated by convection, impacts information encoding and transmission. Convection generates intricate neurotransmitter gradients that influence neural signal interpretation and the mutual information between signals.

Mutual information increases with any rate of neurotransmitter clearance but reaches a plateau at higher rates. Clearance rate variations do not affect the lag time between locations, indicating persistent correlation despite different clearance efficiencies.

## Figures and Tables

**Table 1:** Parameters, used in the model.

Parameter	Units	Values	Meaning
$D_{cleft}$	nm^2^/μs	760	diffusion in cleft, recalculated from [20]
$D_{pm}$	nm^2^/μs	297	diffusion in porous medium, recalculated from [20]
$R_{tot}$	nm	1000	modeling area radius
$R_{cleft}$	nm	200	active synaptic area
$R_{pm}$	nm	400	distance from synaptic center to the end of transition zone
$\Omega_{cleft}$	nm/μs	0–1.9	variable

## Data Availability

The original data presented in the study are openly available in a GitHub repository as Python code that was used in simulations https://github.com/MartyBordeaux/NeuroPhysiology/tree/33bb58dbd421700c5e2e6fa8b9cf5a23c477384e/SynapsePDE (accessed on 13 July 2024).
